# A demographic assessment of the Lansing Effect in duckweed (*Lemna turionifera* Landolt)

**DOI:** 10.1098/rsbl.2024.0271

**Published:** 2024-10-16

**Authors:** Priyanka Dutt, Robert A. Laird

**Affiliations:** ^1^ Department of Biological Sciences, University of Lethbridge, Lethbridge, Alberta T1K 3M4, Canada

**Keywords:** aquatic plants, demography, Lansing Effect, Lemnoideae, lifespan

## Abstract

The Lansing Effect is the propensity for offspring of older parents to have shorter lifespans than offspring of younger parents. A recent review identified two demographic patterns that can produce the Lansing Effect: (i) a greater offspring mortality rate at all offspring ages in offspring of older versus younger parents (greater initial mortality parameter); and (ii) an offspring mortality rate that increases more rapidly with offspring age in offspring of older versus younger parents (greater mortality rate parameter). Here, we report on a longitudinal study designed to investigate these patterns, using the duckweed *Lemna turionifera*. We tracked asexually produced offspring that detached from parents that were comparatively young versus old (first versus fifth offspring, respectively). Offspring of older parents lived 15% shorter, on average, than offspring of younger parents, providing evidence of the Lansing Effect. A model-selection approach revealed that the difference between survival curves of first versus fifth offspring was mainly attributable to greater initial mortality in fifth compared to first offspring, though alternative models also received some support. Our study provides a demographic explanation for the Lansing Effect in *L. turionifera* in particular and provides a method for assessing the survival patterns underpinning the Lansing Effect in general.

## 1. Introduction

The Lansing Effect is the propensity for offspring of older parents to have shorter lifespans than offspring of younger parents [1–3]. This phenomenon, which is a type of parental age effect, has been detected in many species, including humans [4,5] and other vertebrates [6], rotifers [7,8], insects [1,9], nematodes [10] and plants [11–13]. A recent review by Monaghan *et al*. [2] distinguished between two non-exclusive demographic processes that can lead to the Lansing Effect: (i) a greater offspring mortality rate at all offspring ages in offspring of old versus young parents (greater initial mortality parameter); and (ii) an offspring mortality rate that increases more rapidly with offspring age in offspring of old versus young parents (greater mortality rate parameter).

Understanding the contributions of these demographic processes is important for at least two reasons. First, the relative importance of these processes may reflect what makes offspring of older and younger parents different from one another [2]. Specifically, a greater initial mortality for offspring of older parents suggests increased offspring ‘frailty’ that persists but does not worsen with age, while a more rapidly increasing mortality for offspring of older parents suggests a reduced capacity to counteract physiological mechanisms of senescence. Second, the precise nature of parental age effects—such as the Lansing Effect—has potentially important consequences for understanding wider issues such as population age structure, population dynamics, and the evolution of senescence [14,15].

Despite the desirousness of a more in-depth understanding of the Lansing Effect, only a small number of studies have addressed whether offspring of older parents live shorter owing to greater initial mortality, more rapidly increasing mortality, or some combination of the two [2]. Even among these few studies, there is considerable diversity in outcome (see table 1 in [2]). For example, in the fruit fly *Drosophila melanogaster*, increased initial mortality was typically the main cause of the Lansing Effect in daughters of older mothers (though results were strain-dependent) [1]. Conversely, in the crustacean *Daphnia pulex*, a faster rate of ageing was more strongly implicated [16]. The situation was more complicated in the rotifer *Brachionus manjavacas*—offspring of older mothers had increased initial mortality, but the gap narrowed with offspring age [17]. To help expand upon and clarify these advances, additional data on the demographic underpinnings of the Lansing Effect are needed [2], as is greater taxonomic coverage of the topic, particularly in non-animals, mirroring the situation in senescence research in general [18,19].

**Table 1.** Model selection for survival-versus-age for first and fifth offspring, taken separately. Within each birth order, models are given in ascending order of ∆AIC_C_, with the logistic model deemed the best fit in both cases. For parameter estimates, see electronic supplementary material, table S2.

birth order	model	parameters (*k*)	AIC_C_	∆AIC_C_	AIC_C_ weight
first	logistic	3	1145.83	0.00	>9.99 × 10^−1^
	Weibull	2	1225.72	79.89	4.49 × 10^−18^
	Gompertz	2	1294.53	148.70	5.14 × 10^−33^
	exponential	1	1627.69	481.86	2.32 × 10^−105^
fifth	logistic	3	1146.31	0.00	>9.99 × 10^−1^
	Weibull	2	1211.73	65.42	6.24 × 10^−15^
	Gompertz	2	1292.42	146.10	1.88 × 10^−32^
	exponential	1	1572.53	426.21	2.81 × 10^−93^

To this end, we report the results of a longitudinal study designed to investigate the two general demographic processes that can lead to the Lansing Effect, using the duckweed *Lemna turionifera*, a tiny, clonally reproducing aquatic plant, which is a member of a genus subjected to long-standing interest regarding the effects of parental age [12,13,20–23]. Owing to *L. turionifera*’s small stature and ease of cultivation, it allows for large sample sizes, which is advantageous when attempting to distinguish potentially subtle differences in ageing trajectories [24]. We compared the first and fifth offspring, with birth order as a proxy for parental age. While *L. turionifera* frequently has more than 10 offspring under similar conditions to those in our current study [12,13], such that the parents of fifth offspring are not particularly ‘old’ in absolute terms, an analysis of data from a previous study [12,13] leads to the prediction that fifth offspring would have a significantly shorter average lifespan than first offspring, and were therefore suitable for our needs.

Although our primary focus is the Lansing Effect, we also take the opportunity to investigate other parental age effects, including those affecting aspects of offspring quality more closely tied to offspring fitness [20], such as lifetime reproduction, the timing of reproduction, and the intrinsic rate of increase measured at the level of the individual [25]. These fitness-associated parental age effects are important because they are likely to have a greater impact than lifespan variation on the evolution of senescence [14,15]. Moreover, just as in the case of the Lansing Effect, there is considerable diversity in how fitness-associated parental age effects play out [2], including cases in which the shorter-lived offspring of older parents grow faster and reproduce earlier in life compared to the longer-lived offspring of younger parents (i.e. parental age effects on lifespan and reproduction need not go in the same direction; e.g. [16]). An analysis of data from a previous study on *L. turionifera* [12,13] leads to the predictions that fifth offspring would have fewer total offspring themselves, compared to first offspring, but would start reproducing earlier in life, leading to a greater intrinsic rate of increase measured at the level of the individual.

## 2. Methods

### (a) Study species

*Lemna turionifera* Landolt (family Araceae, subfamily Lemnoideae), ‘turion duckweed’, is a small, floating monocot (see electronic supplementary material, appendix S1 for strain details). Each ramet is composed of a single frond (a modified stem–leaf structure) with a single root. Reproduction is predominantly asexual (solely asexual in this study), with offspring plants detaching alternatingly from their parent plant’s two (right and left) meristematic pockets [26].

### (b) Longitudinal study approach

We removed *n* = 392 ‘progenitor plants’ from the working culture and placed them, individually, into sterile 60 mm petri dishes containing 10.5 ml of growth medium (see electronic supplementary material, appendix S1 for growth medium details). Each plant was marked with diluted, autoclaved India ink to make it possible to distinguish parent and offspring plants. Each progenitor plant was randomly assigned to a position on one of 14 wire trays, spread across two growth chambers (Conviron E15; Controlled Environments Limited, Winnipeg, MB, Canada) at 24°C, a photoperiod of 15 : 9 h light : dark, and photon flux densities—measured during the light phase at the start of the study—of 269 μmol m^–2^ s^–1^ for the first chamber and 259 μmol m^–2^ s^–1^ for the second chamber. The 14 wire tray positions were re-randomized daily to mitigate any variation in conditions within and between growth chambers.

Each progenitor plant and its descendants underwent a series of clonal reproductions to establish ‘focal plants’ that were used in the study (electronic supplementary material, figure S1; see electronic supplementary material, appendix S2 for rationale and approach). Half of these were first offspring and half were fifth offspring, allocated randomly. The mean age (± s.e.m.) of parents for which the first offspring was selected was 4.49 ± 0.15 days at the time those first offspring were born; the mean age (± s.e.m.) of parents for which the fifth offspring was selected was 12.07 ± 0.20 days at the time those fifth offspring were born.

Each focal plant was observed daily from birth, defined as the day the focal plant detached from its parent, until death, defined as the day the focal plant’s last offspring detached (determined retroactively following a 7-day buffer period to ensure no further offspring were produced). Thus, death is operationally defined to be the cessation of reproduction, which is an evolutionarily relevant definition in species with no post-birth parental care [27]. Moreover, there are few characteristics that can accurately pinpoint physiological death, making this ‘cessation of reproduction’ definition of death a more consistent and quantifiable approach. During the daily observations, we recorded and discarded offspring of the focal plants.

We removed nine samples from the study owing to microbial contamination or plant damage. The final sample size was *n* = 383 (*n*_first_ = 191; *n*_fifth_ = 192).

### (c) Data analysis

For each focal plant, we measured or calculated the reproductive lifespan (hereafter ‘lifespan’; number of days between birth and cessation of reproduction), age at first reproduction (number of days between the focal plant’s birth and the birth of its first offspring), total number of offspring produced, and intrinsic rate of increase (*r*) measured at the level of the individual; the last of these is the natural logarithm of the leading eigenvalue of a Leslie matrix constructed for a single individual [25]. While lifespan was of primary interest for this study about the Lansing Effect, the other variables were also of interest because they are more closely tied to fitness and, therefore, relevant to understanding the impact of parental age effects on the evolution of senescence [28]. As these variables had skewed distributions, we used randomization tests to compare them between first and fifth offspring (see electronic supplementary material, appendix S3 for details). We estimated the confidence intervals of the differences in mean lifespan, age at first reproduction, total number of offspring produced, and *r* using a bootstrap procedure (see electronic supplementary material, appendix S4 for details).

**Figure 1.**
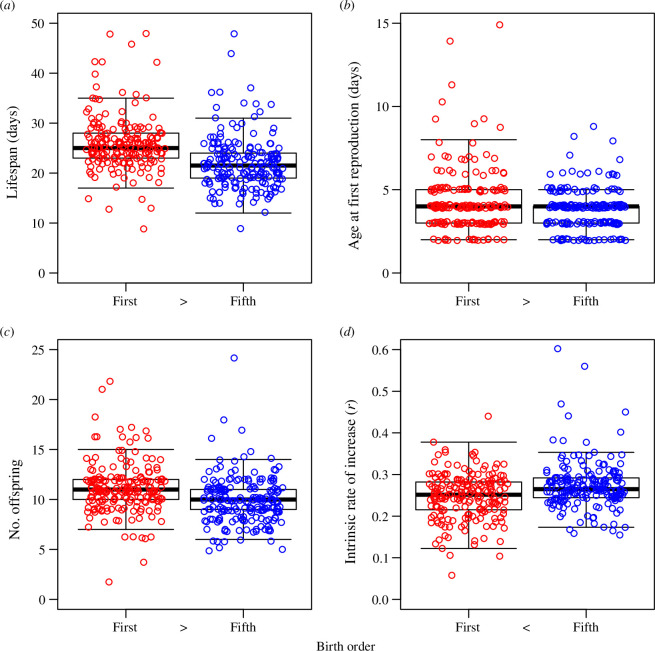
(*a*) Lifespan, (*b*) age at first reproduction, (*c*) number of offspring, and (*d*) intrinsic rate of increase (*r*) for first (red) versus fifth (blue) offspring. In the box-and-whisker plots, thick lines represent medians, the bottom and top edges of the boxes represent the first and third quartiles, respectively, and the lower and upper whiskers represent the smallest and largest values, respectively, that were within 1.5 times the interquartile range of the boxes’ edges. To make the points easier to distinguish, points were subjected to uniform random horizontal jitter; for panels (*a*–*c*), points were also subjected to uniform random vertical jitter (maximum extent of ± 0.3). Randomization tests indicated significant differences between first and fifth offspring for all four response variables; between the birth order labels (i.e. ‘first’ and ‘fifth’), greater than symbols (‘>’) and less than symbols (‘<’) indicate the direction of statistical significance.

A main point raised by Monaghan *et al*.’s [2] verbal model is that if two groups differ in mean lifespan, their mortality trajectories (or, equivalently, survival trajectories) must also differ. The challenge then is to accompany the verbal model with a statistical approach, especially in light of the fact that mortality rates do not normally increase linearly, as in Monaghan *et al*.’s fig. 1, but typically accelerate in early age classes and sometimes plateau in late ones [29,30]. To assess how any differences in lifespan between first and fifth offspring might be related to the nature of their survival curves, we used a two-stage approach. In the first stage, we fit four common survival models for first and fifth offspring, taken separately. The four models were the exponential, Gompertz, Weibull, and logistic models (electronic supplementary material, table S1) [28,30,31]. We did this by maximizing the models’ log-likelihood functions using the *optim()* function in R v. 4.4.0 [32], and estimated the confidence intervals for the fit parameters using a bootstrap procedure by fitting the models to 10 000 bootstrap replicates in which the lifespan distributions of first and fifth offspring were sampled with replacement. We then selected the best model as the one with the lowest Akaike information criterion corrected for small sample sizes (AIC_C_).

Both first and fifth offspring were best fit by the logistic model (see §3), whose survival ( l(t) ) as a function of age ( t ) is given as follows (electronic supplementary material, table S1):


(2.1) $$l\left(t\right)={\left(1+\frac{ac}{b}\left(\exp\left(bt\right)-1\right)\right)}^{-1/c}$$

where a , b and c are the initial mortality parameter (which determines the mortality at age zero), the mortality rate parameter (which affects how quickly mortality changes with age), and the heterogeneity parameter (which reflects variation among individuals), respectively—with the last of these alternately conceptualized as the deceleration parameter describing late-life mortality plateaus [30].

The primacy of the logistic model in the first stage facilitated the second stage of analysis. In this stage, we analysed the survival of first and fifth offspring together using a suite of candidate logistic models. In these candidates, the parameters of the logistic model were either common to first and fifth offspring or distinct between first and fifth offspring. As the logistic model has three parameters, each with two options (common versus distinct), there was a total of 2^3^ = 8 candidate models. As with the first stage, we maximized the candidate logistic models’ log-likelihood functions using *optim*(), estimated confidence intervals via bootstrapping, and selected the best model as the one with the lowest AIC_C_.

## 3. Results

On average, first offspring lived 3.96 days older than fifth offspring (figure 1*a*; 95% confidence interval: [2.89, 5.04]). This difference was significant (randomization test: *p* < 0.0001, *n*_first_ = 191, *n*_fifth_ = 192), confirming the presence of the Lansing Effect.

On average, the age at first reproduction was 0.49 days older for first compared with fifth offspring (figure 1*b*; 95% confidence interval: [0.17, 0.81]), a significant difference (randomization test: *p* = 0.0013, *n*_first_ = 191, *n*_fifth_ = 192).

First offspring had 1.24 more offspring themselves, on average, compared with fifth offspring (figure 1*c*; 95% confidence interval: [0.74, 1.72]), a significant difference (randomization test: *p* < 0.0001, *n*_first_ = 191, *n*_fifth_ = 192). However, owing to first offspring’s longer latency to start reproducing (figure 1*b*) and the fact that first offspring’s offspring were spread out over a longer lifespan (figure 1*a*), first offspring had, on average, a significantly lower intrinsic rate of increase (*r*) compared with fifth offspring (figure 1*d*; difference in means: 0.026; 95% confidence interval: [0.015, 0.037]; randomization test: *p* < 0.0001, *n*_first_ = 191, *n*_fifth_ = 192). We note that this accords with previous work on this strain of *L. turionifera* [12,13], which also demonstrated greater *r* in fifth versus first offspring (referred to therein as third versus first right-meristem offspring), but that *r* decreased substantially in successively higher birth-order offspring.

**Figure 2.**
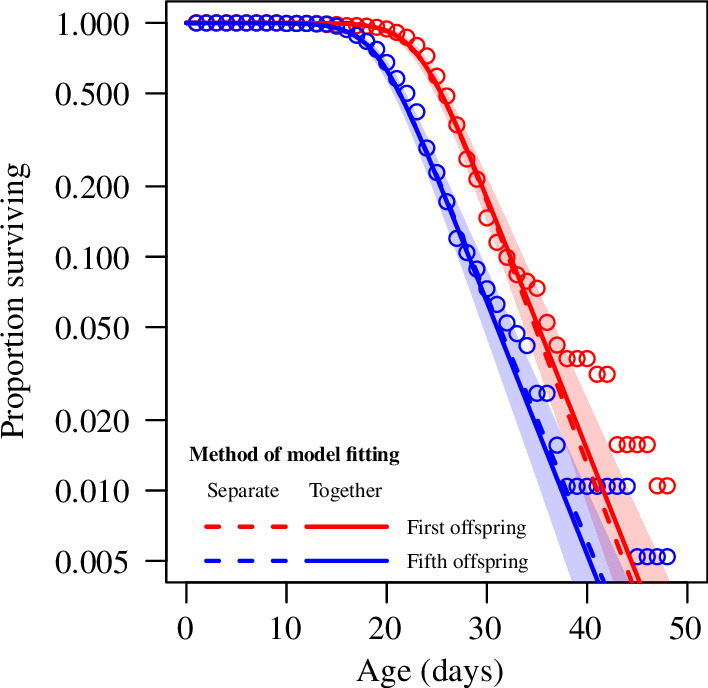
Proportion of plants surviving versus age for first (red) versus fifth (blue) offspring. Note the logarithmic vertical axis. Dashed lines represent logistic model fits for first and fifth offspring taken separately. Solid lines represent the logistic model fit for first and fifth offspring taken together; specifically, for the best-fitting model that had distinct initial mortality parameters but common rate and heterogeneity parameters between first and fifth offspring (shaded regions represent 95% confidence bands).

When fit separately, the survival curves of first and fifth offspring were both best fit with logistic models (table 1; figure 2; electronic supplementary material, table S2 and figure S2). When the survival curves of first and fifth offspring were fit simultaneously, a logistic model with distinct parameter *a* values (i.e. distinct initial mortality, which was almost 10-fold greater in fifth compared with first offspring; electronic supplementary material, table S3), and common parameter *b* and *c* values, was the best fit (table 2; figure 2). Whether survival curves were fit separately or together for first and fifth offspring, they appeared to fit better for plants in the 95th percentile of lifespan in their respective groups and less well for exceptionally long-lived individuals (figure 2). None of the other candidate models had ∆AIC_C_ < 2, which would have indicated similarly strong fits; however, five other candidate models had ∆AIC_C_ < 7 (table 2; electronic supplementary material, figure S3), indicating that they should not be dismissed out of hand [33]. The worst-fit model, with ∆AIC_C_ of 77.95, was the one in which all three logistic parameters were common between first and fifth offspring (table 2; electronic supplementary material, figure S3).

**Table 2.** Model selection for survival-versus-age for first and fifth offspring, taken together. All models are candidate variants of the logistic model and are given in ascending order of ∆AIC_C_, with the Abc model deemed the best fit. Within each model, capital letters in the model name indicate parameters that had distinct values for first and fifth offspring, whereas lower-case letters indicate parameters that had common values for first and fifth offspring. For parameter estimates, see electronic supplementary material, table S3.

model name	status of logistic parameters			parameters (*k*)	AIC_C_	∆AIC_C_	AIC_C_ weight
	a	b	c				
Abc	distinct	common	common	4	2288.29	0.00	4.61 × 10^−1^
ABc	distinct	distinct	common	5	2290.31	2.02	1.68 × 10^−1^
AbC	distinct	common	distinct	5	2290.46	2.17	1.56 × 10^−1^
aBC	common	distinct	distinct	5	2291.03	2.74	1.17 × 10^−1^
ABC	distinct	distinct	distinct	6	2292.11	3.82	6.82 × 10^−2^
aBc	common	distinct	common	4	2293.77	5.48	2.98 × 10^−2^
abC	common	common	distinct	4	2358.02	69.73	3.33 × 10^−16^
abc	common	common	common	3	2366.24	77.95	5.45 × 10^−18^

## 4. Discussion

Our study confirms the existence of the Lansing Effect in duckweeds (figure 1*a*) [11–13] and provides strong evidence for the role of increased initial mortality in offspring of older parents compared with those of younger parents, with the four candidate logistic models that had distinct initial mortality parameters comprising the first-, second-, third- and fifth-highest ranked models (parameter *a*; table 2; electronic supplementary material, figure S3). In each of those models, *a* was greater for fifth offspring by at least fivefold compared with first offspring (electronic supplementary material, table S3). This suggests that the offspring of older parents start life in a frailer condition than those of younger parents. Less clear are the roles of the mortality rate parameter and the heterogeneity/deceleration parameter (parameters *b* and *c*, respectively). The best-fitting logistic model was the one for which these were common between offspring of younger and older parents; however, other models for which ∆AIC_C_ < 7 were inconsistent with respect to those parameters’ distinctiveness (table 2) and inconsistent about whether they were greater for first or fifth offspring (electronic supplementary material, table S3). Although our sample size was large (*n* = 383), it is evident that even larger samples will be required to completely disentangle the candidate models and, therefore, the age-specific differences between offspring of old versus young parents.

Another caveat for our study is that to be able to fit survival curves for discrete categories of offspring of younger versus older parents, it was necessary to use birth order as a proxy for parental age. While birth order is a good proxy for parental age in duckweeds (e.g. the minimum correlation coefficient between birth order and parental age across the offspring of the 383 focal plants in our study was 0.79, and 349 (91%) of the plants had a correlation coefficient greater than 0.95), naturally there is not a one-to-one correspondence. Birth order effects, *per se*, are of interest in their own right [34], and future research should consider how parental age and birth order jointly affect offspring survival trajectories in duckweeds and other taxa.

Our study focused on the demographic underpinnings of the Lansing Effect. An important step forward will be to investigate the physiological underpinnings as well [35,36]. The importance of increased initial mortality in offspring of older parents suggests these clonally produced offspring may have started life with cellular damage acquired by their parents during their lifetimes, which was then transmitted through a mechanism of non-genetic inheritance [12,13,28,37]. Determining the nature of this damage and its transmittance is an important avenue for understanding parental age effects and other cross-generational aspects of senescence in duckweeds. More broadly, from a cross-species comparative perspective, it is important to note that just as there are multiple patterns of offspring survival trajectories that feed into the Lansing Effect [2], the physiological processes are likely similarly diverse.

The Lansing Effect falls within the broader category of parental age effects [1,2]. In addition to having shorter lifespans, fifth offspring in our study also had fewer offspring themselves, compared with first offspring (figure 1*c*), a phenomenon long known to occur in *Lemna* [22]. While our study was predominantly focused on lifespan, investigating how total reproductive output is related to parental age-related patterns of offspring age-specific reproduction should be a priority for future research (e.g. [38]).

Likewise, there is increasing interest in studying parental age effects on offspring traits that reflect both the magnitude and timing of reproduction and are, therefore, more closely related to fitness (e.g. [5,14,15,28,39]). In our study, the intrinsic rate of natural increase measured at the level of the individual (*r*) was actually greater in fifth versus first offspring (figure 1*d*), owing to the former’s shorter time to start reproducing (figure 1*b*) and the fact that their offspring production occurred over a shorter lifespan (figure 1*a*). Whether and how this faster development trades off with capacity for survival is an important open question. Moreover, as with total reproduction, future studies should consider precisely how parental age-associated variation in offspring survival and reproduction trajectories affects measures of fitness. In *Lemna*, such studies should consider higher birth orders relative to total offspring, as the greater *r* we observed in fifth offspring is likely to be reversed in higher-order offspring (e.g. [12,13,28]).

In conclusion, we amplify calls for increased focus on the Lansing Effect, and parental age effects in general, as a means of promoting progress toward understanding the evolution of senescence [14,15]. Uncovering how and why parental age alters age-specific survival and reproduction in offspring—and, in particular, why this differs among taxa [2]—remains an important goal.

## Data Availability

Data and code are available from the Dryad Digital Repository: [40]. Supplementary material is available online [41].
